# Effect of the Polymeric Stabilizer in the Aqueous Phase Fischer-Tropsch Synthesis Catalyzed by Colloidal Cobalt Nanocatalysts

**DOI:** 10.3390/nano7030058

**Published:** 2017-03-06

**Authors:** Jorge A. Delgado, Carmen Claver, Sergio Castillón, Daniel Curulla-Ferré, Cyril Godard

**Affiliations:** 1 Chemical Center of Technology, 43007 Tarragona, Spain; jorge.delgado@ctqc.org; 2 Physicochemical and Inorganic Chemistry Department, Universitat Rovira I Virgili, 43007 Tarragona, Spain; 3 Analytical and Organic Chemistry Department, Universitat Rovira I Virgili, 43007 Tarragona, Spain; sergio.castillon@urv.cat; 4 Total Research and Technology Feluy, B-7181 Seneffe, Belgium; daniel.curulla-ferre@total.com

**Keywords:** aqueous phase Fischer-Tropsch synthesis, cobalt nanoparticles, polymeric stabilizers

## Abstract

A series of small and well defined cobalt nanoparticles were synthesized by the chemical reduction of cobalt salts in water using NaBH_4_ as a reducing agent and using various polymeric stabilizers. The obtained nanocatalysts of similar mean diameters (ca. 2.6 nm) were fully characterized and tested in the aqueous phase Fischer-Tropsch Synthesis (AFTS). Interestingly, the nature and structure of the stabilizers used during the synthesis of the CoNPs affected the reduction degree of cobalt and the B-doping of these NPs and consequently, influenced the performance of these nanocatalysts in AFTS.

## 1. Introduction

Nowadays, nanomaterials afford an unprecedented control of size, shape, and structure at the nanometer scale. The ability to tune nanoparticles and compare series of materials prepared in a similar manner provides a valuable method to obtain information on these catalytic systems working at the molecular and atomic levels [1]. For this reason, metal nanoparticles are a valuable tool for the study of structure sensitive reactions such as the Fischer-Tropsch synthesis (FTS) [2]. Although this reaction has been researched for more than a century, there is a renewed interest for this process since it can provide ultra-clean fuels (nitrogen and sulfur-free) from abundant resources such as natural gas, biomass, and coal [3]. Impregnation methods are usually utilized for the preparation of classical FTS catalysts, although they hardly offer the size and shape modulation that colloidal chemistry does [4]. Metal colloids tend to agglomerate in solution, and thus require the use of capping agents to provide steric or electrostatic stabilization [5]. However, the stabilizing agent is usually not innocent and induces steric restrictions or electronic effects that can affect the performance of the NPs in catalysis [6].

Recent studies have dealt with this effect on FTS catalyzed by RuNPs [7,8]. For instance, Hensen and co-workers reported the deposition of the organic stabilizing agents trimethyl(tetradecyl)ammonium bromide (TTAB), polyvinylpyrrolidone (PVP), and sodium 3-mercapto-1-propanesulfonate (SMPS) onto a previously prepared Ru/CNF (carbon nanofiber) catalyst with the aim of maintaining the size and morphology of these particles. They observed that the activity in the FTS decreased in the following order: Ru > Ru-TTAB > Ru-PVP >> Ru-SMPS and correlated these results with the interaction strength between the organic stabilizing agent and the Ru surface [8]. Very recently, Chaudret and co-workers studied the reactivity of ligand-capped ruthenium nanocatalysts (PVP or diphosphine ligand) in model FTS reactions [7]. They reported that the surface modification by ligands, whether introduced as protecting agents during synthesis or afterwards via ligand exchange, affected both selectivity and activity in FTS catalysis. Indeed, the NPs stabilized by the diphosphine ligand were shown to be more active with a higher selectivity towards light hydrocarbons (C_2_–C_4_) in comparison with the PVP-stabilized RuNPs. In a recent contribution, our group also reported the effect of polymeric stabilizers in the Fischer-Tropsch synthesis catalyzed by TiO_2_-supported cobalt nanoparticles, where the catalytic performance of the catalysts were dependent on the nature of the polymeric stabilizer [9]. Parameters such as the catalyst reducibility, the amount of the polymeric stabilizer remaining at the metal surface, and the metallic cobalt content at the nanoparticle surface were considered to rationalize the performance of these supported catalysts.

In the case of colloidal cobalt catalysts in the AFTS (aqueous phase Fischer-Tropsch synthesis), although several polymers such as PVP [10,11,12] and ionic liquids have been employed as stabilizers [13,14,15], no rational study on the effect of the stabilizer in this reaction has been reported to date.

Kou and co-workers have reported on the application of CoNPs [16] and bimetallic Pt-Co NPs [11], stabilized by PVP, as catalysts of the AFTS. These authors also described the effect of the reducing agent (LiBEt_3_H and NaBH_4_) used for the preparation of CoNPs in the AFTS [10], and proposed that doping by boron could affect the catalytic performance of these NPs. Very recently, Chaudret and co-workers reported the preparation of bimetallic Fe@FeCo and Fe@Ru NPs by thermal decomposition of Co_2_(CO)_8_ or Ru_3_(CO)_12_ carbonyls in the presence of preformed FeNPs [17]. The authors demonstrated the possibility of performing Fischer-Tropsch syntheses by heating the catalytic nanoparticles employing an external alternating magnetic field. Dupont and co-workers reported the synthesis of Co nanocubes (54 ± 22 nm) [13] and nanospheres (7.7 ± 1.2 nm) [15] by thermal decomposition of Co_2_(CO)_8_ in [DMI][NTf_2_] (1-*n*-decyl-3-methylimidazolium *N*-bis(trifluoromethanesulfonyl)-imidate) and [BMI][NTf_2_] (1-*n*-butyl-3-methylimidazolium *N*-bis(trifluoromethanesulfonyl)-imidate), respectively. In catalysis, a higher turnover frequency (TOF) in FTS was observed with the spherical CoNPs in comparison to the nanocubes (0.26 vs. 1.17 × 10^−5^ mol_CO_ mol_Suf-Co_^−1^ h^−1^). Interestingly, the hydrocarbons formed in the FTS using cobalt nanocubes showed a monomodal hydrocarbon distribution centered at C_12_, which was quite different from the bimodal distribution (centered at C_12_ and C_21_) obtained with the spherical cobalt nanoparticles. The authors proposed that these differences were related to the particle-size distributions and to the presence of different active sites (face centered cubic, Co-fcc phase in [BMI][NTf_2_] and ε-phase in [DMI][NTf_2_] stabilized NPs).

In the present work, the effect of the polymeric stabilizer on the structure and catalytic performance in FTS of colloidal cobalt nanoparticles was investigated. For this purpose, a series of CoNPs of similar sizes was prepared by chemical reduction in the presence of several water soluble polymeric stabilizers and was subsequently tested in the aqueous phase Fischer-Tropsch synthesis. 

## 2. Results and Discussion

### 2.1. Synthesis and Characterization of CoNPs

**Figure 1:**
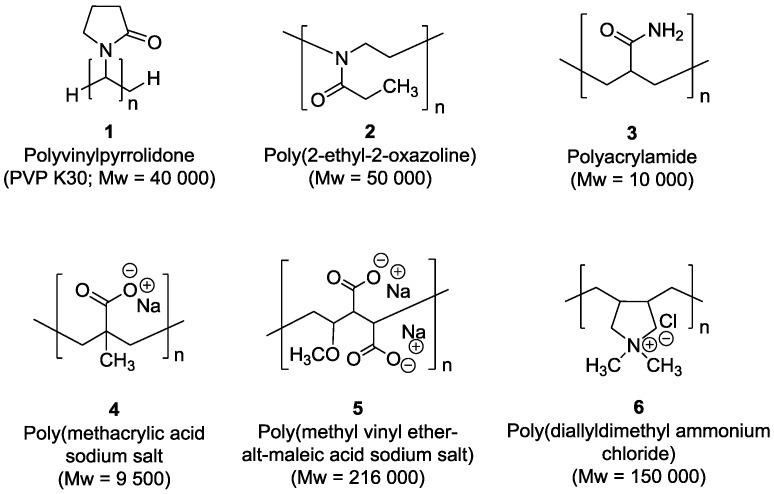
Structures of the polymers (**1**–**6**) used as stabilizers for the CoNPs.

A series of cobalt nanoparticles (**Co1**–**Co6**) were synthesized in water by the chemical reduction of cobalt chloride in the presence of polymeric stabilizers and using sodium borohydride as the reducing agent. The structures of the water soluble polymers used to stabilize these CoNPs are displayed in Figure 1.

**Figure 2:**
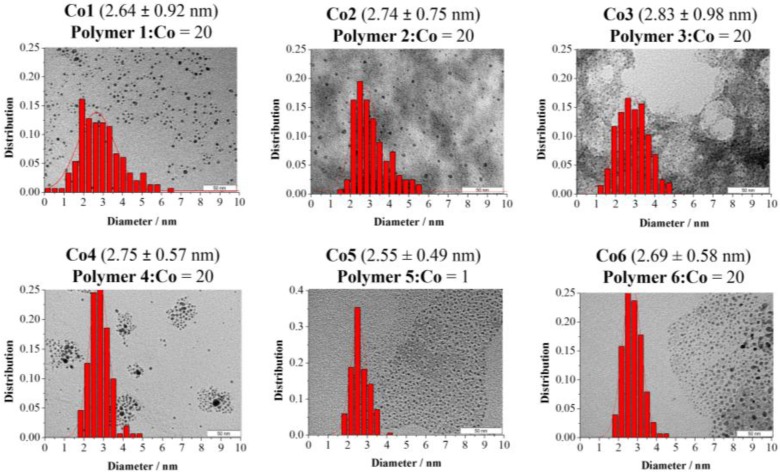
Transmission electron (TEM) micrographs and size histograms of **Co1**–**6** NPs.

**Figure 3:**
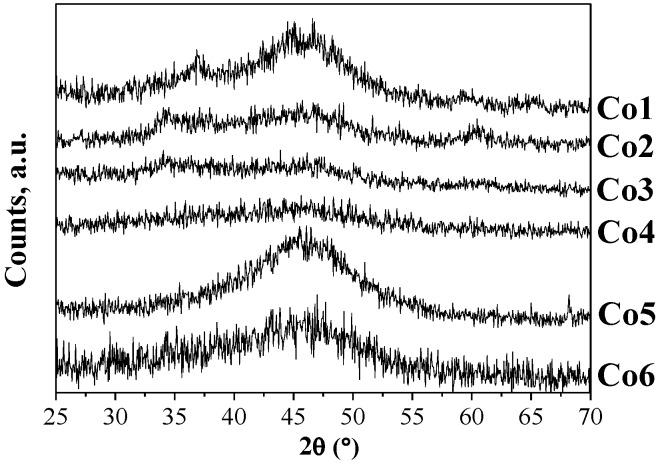
X-ray diffraction patterns of **Co1**–**6** NPs.

To prepare CoNPs of similar size for each one of the polymeric stabilizers, variation of the polymer:Co ratio between 1 and 40 was performed (see Supporting Information) [18,19,20,21]. For most of the polymeric stabilizers, CoNPs of ca. 2.6 nm diameter were produced using a polymer:Co ratio of 20 (**Co1**, **Co2**, **Co3**, **Co4**, and **Co6**). In the case of polymer **5**, NPs of similar size were obtained using a polymer:Co ratio of 1 (**Co5**, 2.55 ± 0.49 nm). The small particle size observed for the series of polymer **5** stabilized NPs at such a low polymer:Co ratio could be attributed to the relatively large molecular weight of this polymer (Molecular weight = 216,000), as previously reported for AuNPs [22]. The transmission electron (TEM) micrographs and size histograms of CoNPs **Co1**–**6** (stabilized by polymers **1**–**6**, respectively) are displayed in Figure 2. Further characterization of these nanoparticles was carried out using X-ray diffraction (XRD), X-ray photoelectron spectroscopy (XPS), Fourier transformed infrared spectroscopy (FTIR), and induced coupled plasma (ICP) analysis. The diffraction pattern of these materials revealed the presence of broad bands at ca. 45°, 35°, and 60° (Figure 3). The broadening of XRD peaks was previously observed for CoNPs prepared by chemical reduction using NaBH_4_ [23,24], and associated to the small size and/or to the amorphous structure of the NPs [25,26]. Surface analysis by X-ray photoelectron spectroscopy (XPS) revealed the presence of Na, Co, O, N, C, and B. Deconvolution of the Co 2p XPS spectra (see Supporting Information) revealed that the metallic cobalt content ranged from 6% to 67% for this series of NPs. **Co5** exhibited the highest content in metallic cobalt (67%) while **Co2**, **Co3**, and **Co4**, presented values of 25%–30%. In the case of **Co6**, only 6% of Co was determined. The differences in the reduction degree of the CoNPs could derive from the capacity of the stabilizing agent to protect the NPs against surface passivation, as previously reported [27,28,29]. For instance, Schmidt and co-workers reported that the oxidation rate of cobalt nanoparticles stabilized by carboxylic acid-telechelic polystyrene (Co@PS) was lower than those stabilized by polycaprolactone (Co@PCL) [28]. According to the authors, this result was explained by the difference in molecular oxygen diffusion resulting from the polarity and oxygen affinity of these polymers.

Furthermore, important amounts of boron were detected in all the CoNPs (2–13 mol %) (Table 1). As previously reported, boron doping frequently occurs during the synthesis of cobalt nanoparticles prepared by NaBH_4_ reduction [24,30]. The molar ratio between the cobalt and boron at the NPs’ surface revealed that the series of CoNPs can be divided in two groups: those with relatively large cobalt content at the surface, with Co/B ratios above 1 (**Co1**, **Co2**, **Co5**, and **Co6**), and those in which the boron content is superior to that of cobalt (**Co3** and **Co4**). In view of these results, it can be concluded that the boron doping at the metal surface highly depends on the polymeric stabilizer used during the synthesis of the CoNPs. During the synthesis of the CoNPs, part of the NaBH_4_ used is catalytically decomposed by the NPs. This catalytic process could therefore be influenced by the structure of the polymeric stabilizer at the surface of the NPs, and factors such as the decomposition rate could somehow favor the boron doping at the cobalt surface, as previously reported in the literature [31].

Moreover, ICP analysis revealed higher Co/B ratios in the bulk of the CoNPs (2–2.8) (Table 1), meaning that the boron doping mainly takes place at the surface of these NPs. Interestingly, the **Co4** NPs constituted the most extreme case with a Co/B ratio of 0.5 at their surface and 6.4 in the bulk.

The presence of the polymeric stabilizer in the CoNPs was examined by FTIR spectroscopy (see Supporting Information). The characteristic signals of the corresponding polymers were only detected for **Co3**, **Co4**, and **Co6**. Trace amounts of the stabilizer could be present in the other NPs, however it is not detectable by this technique.

To summarize, a series of small and well defined CoNPs stabilized by water soluble polymers were synthesized. In most cases, the particle size converged to a value of ca. 2.6 nm when the polymer:Co ratio was 20. Analysis by XRD suggested the amorphous structure of the CoNPs, and XPS analysis revealed the influence of the polymeric stabilizer on the reduction degree and the Co/B ratio at the metal surface. Furthermore, the presence of the stabilizer at the surface of the NPs after their isolation was only detected for the case of **Co3** and **Co4**, according to the FTIR analysis.

### 2.2. Aqueous Phase Fischer-Tropsch Catalytic Experiments

**Figure 4:**
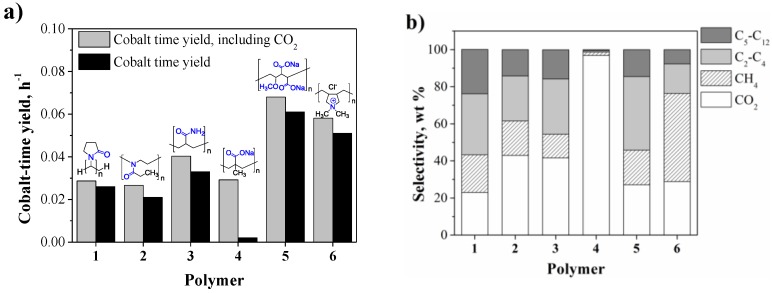
(**a**) Cobalt time yield and (**b**) product selectivity in the AFTS catalyzed by unsupported CoNPs, as a function of the polymeric stabilizer. Conditions: 0.949 mmol Co, 30 bar H_2_/CO/Ar (2:1:0.15), 66 mL water, 1000 rpm, 180 °C, 12 h.

The results corresponding to the AFTS catalyzed by the colloidal nanocatalysts (**Co1**–**6**) are displayed in Figure 4. In terms of activity, moderate cobalt-time yields in the range between 0.002 h^−1^ and 0.061 h^−1^ were obtained for these NPs (Figure 4a). Similar activities were observed for catalysts **Co1** and **Co2** (ca. 0.025 h^−1^), while **Co3** exhibited slightly higher activity (0.033 h^−1^). For the case of **Co4**, although the time yield was almost depreciable, a value up to 0.029 h^−1^ was obtained when the CO converted to CO_2_ was considered in the calculation. Finally, **Co5** and **Co6** exhibited the highest activities of the series with values of 0.061 and 0.051 h^−1^, respectively. The activities obtained in this study are comparable to previous reports on AFTS catalyzed by colloidal cobalt nanoparticles. Kou and co-workers reported activities of ca. 0.10 mol_CO_ mol_Suf-__Co_^−1^ h^−1^ (at 170 °C under 30 bar of syngas in water) for PVP stabilized CoNPs [10,16]. Other studies in which different reaction solvents were employed in FTS are difficult to compare to our aqueous system due to the dependence on mass transfer and syngas solubility as a function of the reaction solvent [23].

Concerning the product selectivity (Figure 4b), large variations were observed with these catalysts. For all the NPs except **Co4**, the CO_2_, CH_4_, C_2_–C_4_, and C_5_–C_12_ selectivities ranged between 23–43, 18–47, 16–40, and 8–24 wt % respectively.

Curiously, **Co4** displayed an almost quantitative selectivity towards CO_2_ (97 wt %). Comparable selectivities in terms of CO_2_, CH_4_, and C_2+_ (ca*.* 24, 20, 55 wt %) were obtained for **Co5** and **Co1** but the former case displayed a higher C_2_–C_4_ fraction (40% vs. 33%) in agreement with a slightly lower α value (0.50 vs. 0.59, respectively). In addition, the olefin/paraffin ratio was remarkably higher for **Co5** compared to that of **Co1** (1.3 vs. 0.8). The catalysts **Co2** and **Co3** exhibited similar CO_2_, CH_4_, and C_2+_ selectivities (ca. 40, 16 and 45 wt %). Finally, a large CH_4_ selectivity (48 wt %) was observed for **Co6** in agreement with a low α value of 0.53. Interestingly the methane selectivity obtained for **Co1** was lower than that reported by Kou and co-workers for the homologous PVP stabilized CoNPs (20% vs. 40%), while the CO_2_ selectivity followed the opposite trend (25% vs. 10%). These differences could be attributed to the distinct particle sizes (2.6 vs. 14 nm) and compositions, based on the Co/B ratio determined for these NPs (2.8 vs. 0.2, respectively).

It is noteworthy that under the current conditions using water as the solvent, the chain length of the hydrocarbon products was not longer than C_9_ (See ASF Distributions in Supporting Information). This particular product distribution is attributed to mass transfer limitations when water is used as the reaction solvent due to the relatively low solubilities of CO and H_2_ in water [23]. From the series of tested catalysts, **Co2** and **Co5** exhibited the lowest and the highest α values (0.50 vs. 0.66, respectively). The rest of the NPs exhibited α values of ca. 0.6 (Supporting Information).

The catalytic performance in FTS of the series of catalysts was rationalized considering mainly the reduction degree and to a lesser extent, the boron and polymer content at the surface of the CoNPs. Additionally, TEM, XPS, and FTIR analysis were performed on the spent catalyst to study changes in size, reduction degree, and composition of the CoNPs during catalysis.

**Figure 5:**
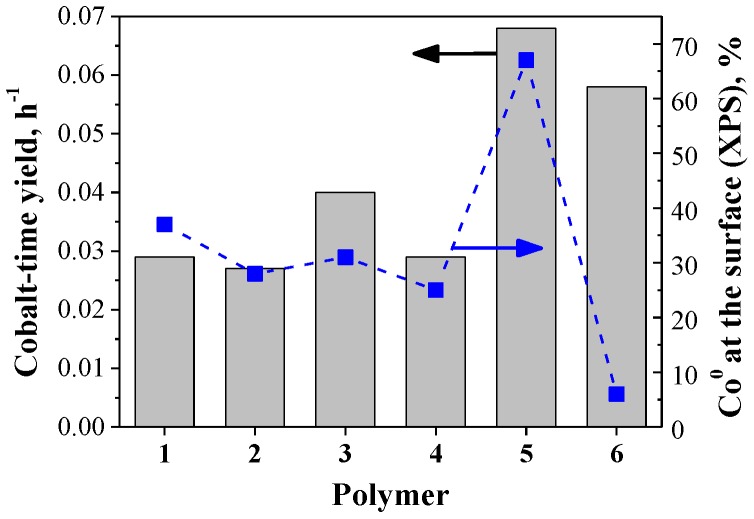
Correlation between the activity and the reduction degree at the surface of the CoNPs as a function of the polymeric stabilizer (Co^0^ determined by XPS).

The reduction degree of the fresh CoNPs is of paramount importance for their activity since in the absence of activation pretreatment, it determines the amount of the active phase at the beginning of the reaction. The relationship between the activity and the reduction degree of the CoNPs is represented in Figure 5. According to this plot, there is a clear correlation between the percentage of reduced cobalt and the activity for the majority of the NPs (**Co1**–**5**), with **Co5** being the most active catalyst of the series. In the case of **Co6**, it is proposed that additional factors must affect the activity of these NPs. For instance, despite the fresh **Co6** NPs presenting a low content of reduced cobalt at the surface, it has been reported that both the presence of chloride (as a counter ion of the polycationic stabilizer, polymer **6**) [32], and the presence of metallic cobalt in the core of the CoNPs [33], promote and assist the metal reduction of the cobalt NPs.

In relation with the reduction degree discussed above, the analysis by XPS of the used catalysts revealed that in most cases, the percentage of reduced cobalt decreased substantially during catalysis (ca. −15% to −65%, see Supporting Information), thus evidencing relevant oxidation of the CoNPs under the current reaction conditions. According to the study reported by van Steen and co-workers on the thermodynamic stability of small cobalt nanoclusters in FTS, it is very likely that the combination of the small particle size of the CoNPs and the use of water as the reaction solvent is responsible for such oxidation [34]. Interestingly, **Co3** maintained its reduction degree during FTS with values of ca. 35%. This observation could be explained by the considerable growth of **Co3** during the reaction (from 2.8 ± 1.0 to 11.6 ± 4.9; see Supporting Information), considering that the bigger CoNPs are less susceptible to oxidation under FTS conditions. The other CoNPs in general exhibited less marked changes in particle size (ca. ±1 nm) during the reaction with respect to the fresh catalyst.

Concerning the effect of boron on catalysis, it has been reported that B-doping could negatively or positively affect the catalytic activity and the reducibility of cobalt based FT catalysts [35,36]. For instance, Kou and co-workers analyzed by ICP the cobalt and boron content of CoNPs prepared from NaBH_4_ and LiBEt_3_H, determining Co/B atom ratios of 0.2 and 2, respectively [10]. They proposed that the large boron content in the Co-NaBH_4_ explained its lower catalytic performance in comparison to Co-LiBEt_3_H considering that the excess of B could noticeably reduce the activity in Fischer-Tropsch syntheses (0.10 vs. 0.27 mol_CO_ mol_Suf-Co_^−1^ h^−1^) [35]. Conversely, studies based on DFT calculations and experimentation demonstrated that boron can act as a promoter to enhance the stability of cobalt based catalysts during the Fischer-Tropsch synthesis [36,37]. Recently, our group reported the unconventional stability of very small cobalt nanocrystals (1.6–7 nm) in FTS due to the promoting effect of boron [38].

**Figure 6:**
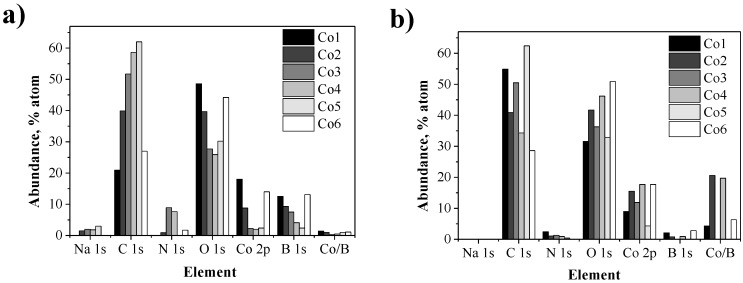
Surface elemental composition of (**a**) fresh and (**b**) used catalysts after AFTS determined by XPS analysis.

With the aim of examining the changes in composition at the metal surface of the CoNPs (e.g., boron content, or the presence of polymeric stabilizers), XPS and FTIR analysis were performed on the used catalysts. The elemental composition of the fresh and used catalysts after AFTS are displayed in Figure 6. The variation in the elemental abundances between the fresh and used catalysts demonstrates a change in composition of the metal surface during the FTS reaction. For instance, trace amounts of sodium (coming from the NaBH_4_) were observed in most of the fresh catalysts but were not detected after catalysis. Similarly, the amounts of B and N decreased substantially (or even disappeared) after catalysis. The decrease of boron at the metal surface after catalysis is evident looking at the Co/B ratio which increased in all the cases up to values of 20. In addition, FTIR analysis of the used catalysts (Supporting Information), evidenced the disappearance of the characteristic signals of the polymeric stabilizer in those cases in which the polymer was detected in the fresh catalysts (**Co3** and **Co4**). The decrease or disappearance of elements such as Na, B, and N as well as of the polymeric stabilizer suggest a process of restructuration of the metal surface during the aqueous phase FTS under the current reaction conditions. These results are in contrast with those reported by Kou and co-workers, that did not observe variations of the Co/B ratio before and after AFTS for borohydride reduced CoNPs [10].

Concerning the product selectivity, **Co4** and **Co6** exhibited high selectivities towards CO_2_ and CH_4_, respectively (97% and 48%). The other catalysts (**Co1**, **Co2**, **Co3**, and **Co5**) exhibited typical product selectivities for the aqueous phase FTS catalyzed by CoNPs [23,24]. The selectivity of **Co4** towards CO_2_ can be correlated to the presence of sodium at the metal surface since it was the counter ion of the polyanionic stabilizer (polymer **4**, Figure 1) [39]. The high methane selectivity observed for **Co6** can be related to the effect of the traces of chloride (detected by XPS, Supporting Information) which was reported to enhance the methanation activity of cobalt based FTS catalysts. According to Burtron and co-workers, the presence of chloride enhances the methane and light hydrocarbon selectivity of a Co/γ-Al_2_O_3_ catalyst, probably due to a blocking effect of metal sites by the halide in agreement with H_2_ chemisorption measurements performed on the used catalysts [40].

To conclude, relevant differences in the catalytic performance of **Co1**–**6** were observed in AFTS depending on the polymeric stabilizer used during the synthesis of the NPs, which mainly affected the reduction degree of the CoNPs. The correlation between the activity and other parameters such as the polymer content and B-doping was less evident because of the possibility of surface restructuration of the NPs under reaction conditions.

## 3. Materials and Methods

### 3.1. Synthesis of Polymeric Stabilized CoNPs, **Co1**–**Co6**

The series of CoNPs, **Co1**–**Co6** were synthesized by chemical reduction of cobalt chloride in the presence of various water soluble polymeric stabilizers and using sodium borohydride as the reducing agent. As a standard procedure, 0.226 g of CoCl_2_·6H_2_O (0.931 mmol) was dissolved in H_2_O containing the corresponding amount of the polymeric stabilizer (polymer:Co ratio varied from 1 to 40). The volume of water for all the syntheses was 50 mL to obtain a cobalt concentration of 0.018 M. Then, a freshly prepared solution of 0.358 g of NaBH_4_ (9.31 mmol) in 16.6 mL H_2_O was added at room temperature at a rate of 3 mL/min (5 min). The solution was maintained under vigorous mechanical stirring for 2 h. Then a sample of the colloidal solution was centrifuged, washed with water, and re-dispersed by sonication. Three drops of the obtained colloidal solution was deposited on a Cu-formvar grid for TEM analysis. For the isolation of the CoNPs, the freshly prepared NPs were initially precipitated by a strong magnetic field and the supernatant was decanted. Then, the precipitated NPs were washed with water to remove the excess salts and polymers. The decantation and washing process was repeated three times with water, ethanol, and hexane. The resulting CoNPs were finally dried under vacuum and kept in the glove box.

### 3.2. Aqueous Phase Fischer-Tropsch Experiments

Catalytic experiments were performed according to reported methods [41], in a 100 mL stirred tank reactor operated in bath mode. A suitable amount of isolated CoNPs (ca. 0.60 mg corresponding to ca. 1 mmol of Co) were re-dispersed in 66 mL of water, and placed inside a Teflon liner in the autoclave. The autoclave was purged three times with Ar, and sealed at an Ar pressure of 1.5 bar. Additionally, 10 bar CO and 20 bar H_2_ were added, giving a final pressure of 31.5 bar (H_2_:CO:Ar = 2:1:0.15). Then the autoclave was heated at 180 °C under mechanical stirring at 1000 rpm for 12 h. After the reaction, the autoclave was cooled to room temperature prior to gas analysis. All the components contained in the gas phase (CO, H_2_, Ar, CO_2_, and C_1–8_ hydrocarbons) were analyzed by gas chromatography (provided with a thermal conductivity detector-TCD) and the quantification was performed using calibration curves for each component. The compounds present in the aqueous phase were extracted with dichloromethane (10 mL) containing 1 µL of bicyclohexyl as an internal standard. The organic phase containing the hydrocarbon and oxygenated products (alcohols and aldehydes) were analyzed by gas chromatography-mass spectromentry (GC-MS). The identification and quantification of products was performed by comparison with standards using calibration curves for each compound.

## 4. Conclusions

A series of small and well defined CoNPs with diameters of ca. 2.6 nm stabilized by water soluble polymers were synthesized. Notable differences in the structure, the oxidation degree, the composition, and the presence of remaining polymer were evidenced.

In the aqueous phase Fischer-Tropsch synthesis (AFTS), the activity of the colloidal nanocatalysts was fairly correlated with the reduction degree of the CoNPs. The excellent performance observed for **Co5** was in agreement with the remarkably high reduction degree of the fresh catalyst. Marked variations in the product selectivity were obtained in CoNPs stabilized by polyelectrolytes. CoNPs stabilized by polymers containing Na^+^ and Cl^−^ (polymer **4** and polymer **6**) as counter ions displayed high selectivities towards the CO_2_ or CH_4_ reaction, respectively.

Characterization of the spent catalysts evidenced variations of the surface composition and the particle size of the CoNPs, thus suggesting some restructuration of the metal surface of the CoNPs during catalysis.

## Figures and Tables

**Table 1:** Elemental quantification by X-ray photoelectron spectroscopy (XPS) and induced coupled plasma (ICP) of **Co1**–**6** NPs.

NPs	Polymer	XPS Quantification Regions (mol %)							ICP
		Na 1s	C 1s	N 1s	O 1s	Co 2p	B 1s	Co/B	Co/B
**Co1**	1		21		49	18	13	1.4	2.8
**Co2**	2	2	40	1	40	9	9	1.0	2.6
**Co3**	3	2	52	9	28	2	8	0.3	2.4
**Co4**	4	2	59	8	26	2	4	0.5	6.4
**Co5**	5	3	62		30	2	2	1.0	2.5
**Co6**	6		27	2	44	14	13	1.1	2.0

## Supplementary Materials

The following are available online at http://www.mdpi.com/2079-4991/7/3/58/s1.

## Abbreviations

The following abbreviations are used in this manuscript:

AFTS	aqueous phase Fischer-Tropsch synthesis
WGS	water gas shift
NPs	nanoparticles
TEM	transmission electron microscopy
XRD	X-ray diffraction
XPS	X-ray photoelectron spectroscopy
FTIR	Fourier-transformed infrared spectroscopy
ICP	indouced coupled plasma
