# Supplementary material for: Effect of the Polymeric Stabilizer in the Aqueous Phase Fischer-Tropsch Synthesis Catalyzed by Colloidal Cobalt Nanocatalysts
Source: Nanomaterials (Basel). 2017 Mar 6;7(3):58. doi: 10.3390/nano7030058 (PMC5388160; doi:10.3390/nano7030058)
Supplement: Supplementary file 1 [file nanomaterials-07-00058-s001.pdf]

Effect of the Polymeric Stabilizer in the Aqueous Phase Fischer-Tropsch Synthesis Catalyzed by Colloidal Cobalt Nanocatalysts

Supporting Information

Content

1. Materials and Methods 2

1.1. Product analysis of Fischer-Tropsch experiments.. 3

1.2. Activity and Selectivity Calculations 3

1.3. Synthesis of colloidal cobalt nanoparticles by chemical reduction (Co1-6) 3

2. XPS analysis of the CoNPs before and after catalysis. 11

2.1. Evolution of the metallic cobalt content at the NPs surface after catalysis..................... 11

2.2. Evolution of the oxidation state of boron species at the NPs surface after catalysis 12

2.3. Complementary XPs analysis (full XPS and C 1s) of the fresh catalysts. 14

3. TEM analysis of the CoNPs after catalysis. 15

4. FTIR spectra of the CoNPs after catalysis. 17

5. Aqueous phase Fisher-Tropsch synthesis (AFTS) data.. 18

1. Materials and Methods

All syntheses of CoNPs were carried under aerobic conditions using a mechanical stirrer. Milli-Q water was used for all the experiments. Solvents were purchased from Merck and used as received. $CoCl_2 \cdot 6H_2O$, $NaBH_4$ and the polymers were purchased from Sigma-Aldrich. Hydrogen (5.0) was purchased from Air Liquide and CO (4.7) and argon (5.0) from Carburos Metálicos.

Transmission electron microscopy (TEM) measurements were performed at the "Unitat de Microscopia dels Serveis Cientificotècnics de la Universitat Rovira I Virgili" in Tarragona with Zeiss 10 CA electron microscope operated at 100 kV with resolution of 3 Å. High resolution electron microscopy (HRTEM) measurements were performed at the "Centres Científics i Tecnològics de la UB" in Barcelona with a JEOL 2011(FEG) electron microscope operated at 200 kV with a point resolution of 2 Å.

XRD measurements were made using a Siemens D5000 diffractometer (Bragg-Brentano parafocusing geometry and vertical θ-θ goniometer) fitted with a curved graphite diffracted-beam monochromator, incident and diffracted -beam Soller slits, a 0.06 º receiving slit and scintillation counter as a detector. The angular 2θ diffraction range was between 25 and 120º. The data were collected with an angular step of 0.05 º at 16s per step and sample rotation. A low background Si(510) wafer was used as sample holder. $Cu_{k\alpha}$ radiation was obtained from a copper X-ray tube operated at 40 kV and 30 mA.

XPS experiments were performed in a PHI 5500 Multitechnique System (from Physical Electronics) with a monochromatic X-ray source (Aluminium Kalfa line of 1486.6 eV energy and 350 W), placed perpendicular to the analyzer axis and calibrated using the 3d5/2 line of Ag with a full width at half maximum (FWHM) of 0.8 eV. The analized area was a circle of 0.8 mm diameter, and the selected resolution for the spectra was 187.5eV of Pass Energy and 0.8 eV/step for the general spectra and 23.5 eV of Pass Energy and 0.1 eV/step for the spectra of the different elements in the depth profile spectra. A low energy electron gun (less than 10 eV) was used in order to discharge the surface when necessary. All Measurements were made in a ultra high vacuum (UHV) chamber pressure between $5x10^{-9}$ and $2x10^{-8}$ torr.

FTIR spectra were acquired from KBr discs on a Bruker Equinox 55 Spectrometer using Opus software. Raman spectra was acquired by scattering of a 514nm Ar lamp on a Renishaw InVia Raman microscope using a x50LW objective, 60s of exposition time and 100% of laser intensity.

Thermogravimetric analysis (TGA) were performed at the "Centres Científics i Tecnològics de la UB" in Barcelona with a thermobalance (Mettler TGA/SDTA851e) equipped with a gas flow system. A 2-6 mg sample was heated in an alumina crisol in a flow of nitrogen. Then the catalyst was heated under a 50 ml/min of nitrogen flow from 30 ℃ to 900 ℃ (heat rate: 10 ℃/min).

Temperature programmed reduction under H_2 (TPR) was carried out using a ChemBET TPR/TPD (Quantachrome). The catalyst (0.1 g) was mounted in a quartz cell and heated up to 900 ℃ in a flow of 5% H_2/Ar (30 $cm^3 min^{-1}$). The rate of temperature ramp was 10 ℃ min^{-1}.

GC-TCD analyses were carried out on an Agilent 7890A GC using a system with three columns: Hayesep Q (3ft x 1/8 in), HP-Molesieve (30m x 0.320 mm x 12 μm) and HP-Plot/Q (30m x 0.320 mm x 20 μm) using helium as carrier gas. GC-MS analyses were performed on a 9575C MSD system (Agilent) using a HP-Innowax capillary column (30m x 0.250 mm x 0.15 μm), using helium as carrier gas. Standard reference of gases (CO, CO_2, H_2, N_2) and hydrocarbons (C_1-C_5) was purchased from Avello Linde.

1.1. Product analysis of Fischer-Tropsch experiments

All the hydrocarbon products were identified by comparison with reference samples. The components contained in the gas phase (CO, H_2, Ar, CO_2, and C_1-C_8 hydrocarbons) were analysed on a Agilent 7890A GC using a system of three columns: Hayesep Q (3ft x 1/8 in), HP-Molesieve (30m x 0.320 mm x 12 μm) and HP-Plot/Q (30m x 0.320 mm x 20 μm) using helium as carrier gas. The quantification was carried out using calibration curves for each gas. Mixtures at different concentration of gases were prepared by gas blending from a standard reference of gases (Avello Linde).

The compounds present in the aqueous phase were extracted with dichloromethane (10 ml) containing 1 μl of bicyclohexyl as internal standard. The bottom organic phase containing the hydrocarbon and oxygenated products were analysed immediately by GC-MS on a 9575C MSD system using a using a HP-Innowax capillary column (30m x 0.250 mm x 0.15 μm), and helium as carrier gas.

The identification and quantification of products was performed by comparison with standards using calibration curves for each compound.

1.2. Activity and Selectivity Calculations

The activity and selectivity was calculated based on the number of moles of carbon being formed as products according to the following formulas:

$$\text{Cobalt time yield} = \frac{\text{mol of CO converted}}{\text{mol of Co} * \text{reaction time (h)}}$$

$$\text{Selectivity } Cn = \frac{\text{Wt } C_n}{\text{Wt of all products}}\ x\ 100$$

1.3. Synthesis of colloidal cobalt nanoparticles by chemical reduction (Co1-6)

CoNPs were synthesized by chemical reduction of cobalt chloride in presence of different water soluble polymers as stabilizer and using sodium borohydride as reducing agent. As standard

procedure, 0.226 g of CoCl2.6H2O (0.931 mmol) was dissolved in H2O containing the corresponding amount of the polymer stabilizer. The volume of water for all the synthesis was completed to 50 ml to obtain a cobalt concentration of 0.018 M. Then, a freshly prepared solution of 0.358 g of NaBH4 (9.31 mmol) in 16.6 ml H2O was added at room temperature with a rate of 3 ml/min (5 min). The solution was maintained under vigorous mechanical stirring for 2 h. Then 100 µl of the colloidal solution was centrifuged, washed with water and re-dispersed by sonication. Three drops of the obtained colloidal solution was deposited on a Cu-formvar or holey carbon grids for TEM or HR-TEM analysis. For the isolation of the CoNPs, the freshly prepared NPs were initially precipitated by a strong magnetic field and the supernatant was decanted. Then, the precipited NPs were rinsed with water to remove the excess of salts and polymers. The decantation and washing process was repeated three times with water, then with ethanol and hexane. The resulted CoNPs were finally dried under vacuum and kept in the glove box. The size, the crystalline structure and oxidation state of the CoNPs were studied using transmission electron microscopy (TEM), HR-TEM, X-ray diffraction (XRD), and X-ray photoelectron spectroscopy (XPS) respectively. The composition of the CoNPs was studied using FTIR, TGA and ICP-OES.

- **Co1a:** was synthesized according to the general procedure using $CoCl_2 \cdot 6H_2O$ (0.226 g, 0.931 mmol) in presence of **Polymer1** (1.033 g of PVP-K30, **Polymer1**:Co=10) dissolved in 50 ml of deionized water ([Co] = 0.018 M). $NaBH_4$ (0.358 g, 9.369 mmol) was dissolved in 16.6 ml ([$NaBH_4$] = 0.550 M).
  TEM: D = 3.13 ± 1.58 nm.
- **Co1b (labeled in the paper as Co1):** was synthesized according to the general procedure using $CoCl_2 \cdot 6H_2O$ (0.226 g, 0.931 mmol) in presence of **Polymer1** (2.066 g of PVP-K30, **Polymer1**:Co=20) dissolved in 50 ml of deionized water ([Co] = 0.018 M). $NaBH_4$ (0.358 g, 9.369 mmol) was dissolved in 16.6 ml ([$NaBH_4$] = 0.550 M). **TEM:** D = 2.64 ± 0.92 nm.
  HR-TEM: *ca.* 2.6 nm; detection of CoO-fcc and Co_3O_4-fcc.
  XRD: 45, 35 and 60 °
  XPS: Co^0, 37%; Co^{2+}, 63%.
  TGA: Temperature ºC/Δ wt. % (attribution); 70/-3.5 (solvent), 244/-3.7 (polymer), 900end/+0.92.
  ICP: Co, 82.4 wt%, B, 5.9 wt%; Co/B atom ratio, 2.79
  FTIR: 3477, 1644, 1425 cm^{-1}.
  RAMAN: 666, 586, 492, 450, 196 cm^{-1}.
- **Co1c:** was synthesized according to the general procedure using $CoCl_2 \cdot 6H_2O$ (0.226 g, 0.931 mmol) in presence of **Polymer1** (4.132 g of PVP-K30, **Polymer1**:Co=40) dissolved in 50 ml of deionized water ([Co] = 0.018 M). $NaBH_4$ (0.358 g, 9.369 mmol) was dissolved in 16.6 ml ([$NaBH_4$] = 0.550 M). **TEM:** D = 2.11 ± 0.41 nm.

- **Co2a:** was synthesized according to the general procedure using $CoCl_2{\cdot}6H_2O$ (0.226 g, 0.931 mmol) in presence of **Polymer2** (0.432 g of poly(2-ethyl-2-oxazoline), **Polymer2**:Co=10) dissolved in 50 ml of deionized water ([Co] = 0.018 M). $NaBH_4$ (0.358 g, 9.369 mmol) was dissolved in 16.6 ml ([$NaBH_4$] = 0.550 M).

  TEM: D = 6.73 ± 1.58 nm.

- **Co2b (labeled in the paper as Co2):** was synthesized according to the general procedure using $CoCl_2{\cdot}6H_2O$ (0.226 g, 0.931 mmol) in presence of **Polymer2** (0.864 g of poly(2-ethyl-2-oxazoline), **Polymer2**:Co=20) dissolved in 50 ml of deionized water ([Co] = 0.018 M). $NaBH_4$ (0.358 g, 9.369 mmol) was dissolved in 16.6 ml ([$NaBH_4$] = 0.550 M).

  TEM: D = 2.74 ± 0.75 nm.

  XRD: 34, 47 and 61 º

  XPS: Co^0, 28%; Co^{2+}, 72%.

  TGA: Temperature ºC/Δ wt. % (attribution); 70/-7.7 (solvent); 285/-6.7 (polymer); 424/-0.7 (polymer); 900end/+2.74

  ICP: Co, 81.0 wt%, B, 6.3 wt%; Co/B atom ratio, 2.57

  FTIR: 3449, 1635, 1418, 1053 cm^{-1}.

  RAMAN: 670, 605, 508, 464, 184 cm^{-1}.

- **Co2c:** was synthesized according to the general procedure using $CoCl_2{\cdot}6H_2O$ (0.226 g, 0.931 mmol) in presence of **Polymer2** (1.728 g of poly(2-ethyl-2-oxazoline), **Polymer2**:Co=40) dissolved in 50 ml of deionized water ([Co] = 0.018 M). $NaBH_4$ (0.358 g, 9.369 mmol) was dissolved in 16.6 ml ([$NaBH_4$] = 0.550 M).

  TEM: D = 1.84 ± 0.74 nm.

- **Co3a:** was synthesized according to the general procedure using $CoCl_2{\cdot}6H_2O$ (0.226 g, 0.931 mmol) in presence of **Polymer3** (0.659 g of 50 wt% sln. of polyacrylamide, **Polymer3**:Co=10) dissolved in 50 ml of deionized water ([Co] = 0.018 M). $NaBH_4$ (0.358 g, 9.369 mmol) was dissolved in 16.6 ml ([$NaBH_4$] = 0.550 M). D = 3.26±1.12 nm.

  TEM: D = 3.26 ± 1.12 nm.

- **Co3b (labeled in the paper as Co3):** was synthesized according to the general procedure using $CoCl_2{\cdot}6H_2O$ (0.226 g, 0.931 mmol) in presence of **Polymer3** (1.318 g of 50 wt% sln. of polyacrylamide, **Polymer3**:Co=20) dissolved in 50 ml of deionized water ([Co] = 0.018 M). $NaBH_4$ (0.358 g, 9.369 mmol) was dissolved in 16.6 ml ([$NaBH_4$] = 0.550 M).

  TEM: D = 2.83 ± 0.98 nm.

  XRD: 35, 46 and 60 º

  XPS: Co^0, 31%; Co^{2+}, 69%.

  TGA: Temperature ºC/Δ wt. % (attribution); 89/-8 (solvent); 224/-6 (polymer); 318/-8; 434/-12; 524/-8 (polymer); 900end/-6.

  ICP: Co, 56.3 wt%, B, 4.7 wt%; Co/B atom ratio, 2.39

  FTIR: 3396, 3196, 2941, 1664, 1612, 1413 cm^{-1}.

RAMAN: 775, 671 cm^{-1}.

- **Co3c:** was synthesized according to the general procedure using $CoCl_2{\cdot}6H_2O$ (0.226 g, 0.931 mmol) in presence of **Polymer3** (2.635 g of 50 wt% sln. of polyacrylamide, **Polymer3**:Co=40) dissolved in 50 ml of deionized water ([Co] = 0.018 M). $NaBH_4$ (0.358 g, 9.369 mmol) was dissolved in 16.6 ml ([$NaBH_4$] = 0.550 M).

  TEM: D = 1.67 $\pm$0.46 nm.

- **Co4a:** was synthesized according to the general procedure using $CoCl_2{\cdot}6H_2O$ (0.226 g, 0.931 mmol) in presence of **Polymer4** (1.670 g of 50 wt% sln. of poly(methacrylicacid) sodium salt, **Polymer4**:Co=10) dissolved in 50 ml of deionized water ([Co] = 0.018 M). $NaBH_4$ (0.358 g, 9.369 mmol) was dissolved in 16.6 ml ([$NaBH_4$] = 0.550 M).

  TEM: D = 4.12 $\pm$1.58 nm.

- **Co4b (labeled in the paper as Co4):** was synthesized according to the general procedure using $CoCl_2{\cdot}6H_2O$ (0.226 g, 0.931 mmol) in presence of **Polymer4** (3.340 g of 50 wt% sln. of poly(methacrylicacid) sodium salt, **Polymer4**:Co=20) dissolved in 50 ml of deionized water ([Co] = 0.018 M). $NaBH_4$ (0.358 g, 9.369 mmol) was dissolved in 16.6 ml ([$NaBH_4$] = 0.550 M).

  TEM: D = 2.75 $\pm$0.57 nm.

  XRD: Not peaks detected.

  XPS: Co^0, 25%; Co^{2+}, 75%.

  TGA: Temperature ºC/Δ wt. % (attribution); 92/-3.4(solvent); 215/-6.2 (polymer low [386/-3.8; 623/-2.2 (polymer); 900end/+0.26

  ICP: Co, 81.9 wt%, B, 2.6 wt%; Co/B atom ratio, 6.44

  FTIR: 3430, 2923, 1555, 1399, 1205 cm^{-1}.

  RAMAN: 680 cm^{-1}.

- **Co4c:** was synthesized according to the general procedure using $CoCl_2{\cdot}6H_2O$ (0.226 g, 0.931 mmol) in presence of **Polymer4** (6.680 g of 50 wt% sln. of poly(methacrylicacid) sodium salt, **Polymer4**:Co=40) dissolved in 50 ml of deionized water ([Co] = 0.018 M). $NaBH_4$ (0.358 g, 9.369 mmol) was dissolved in 16.6 ml ([$NaBH_4$] = 0.550 M).

  TEM: D = 1.61 $\pm$0.67 nm.

- **Co5a (labeled in the paper as Co5):** was synthesized according to the general procedure using $CoCl_2{\cdot}6H_2O$ (0.226 g, 0.931 mmol) in presence of **Polymer5** (2 ml of 0.375 mM sln. of poly(methylvinylether-alt-maleic acid) sodium salt, **Polymer5**:Co=1) dissolved in 48 ml of deionized water ([Co] = 0.018 M). $NaBH_4$ (0.358 g, 9.369 mmol) was dissolved in 16.6 ml ([$NaBH_4$] = 0.550 M).

  TEM: D = 2.55 $\pm$0.49 nm.

  HRTEM: *ca.* 2.6 nm; detection of CoO-fcc, Co_3O_4-fcc and Co-hcp.

  XRD: 46 º

  XPS: Co^0, 67%; Co^{2+}, 33%.

TGA: Temperature ºC/Δ wt. % (attribution); 114/-0.6 (solvent); 900end/+4.35.

ICP: Co, 75.4 wt%, B, 6.2 wt%; Co/B atom ratio, 2.45

FTIR: 3482 cm^{-1}.

RAMAN: 825 cm^{-1}.

- **Co5b:** was synthesized according to the general procedure using $CoCl_2{\cdot}6H_2O$ (0.226 g, 0.931 mmol) in presence of **Polymer5** (10 ml of 0.375 mM sln. of poly(methylvinylether-alt-maleic acid) sodium salt, **Polymer5**:Co=5) dissolved in 40 ml of deionized water ([Co] = 0.018 M). $NaBH_4$ (0.358 g, 9.369 mmol) was dissolved in 16.6 ml ([$NaBH_4$] = 0.550 M).

  TEM: D = 2.07 ± 0.68 nm.

- **Co5c:** was synthesized according to the general procedure using $CoCl_2{\cdot}6H_2O$ (0.226 g, 0.931 mmol) in presence of **Polymer5** (20 ml of 0.375 mM sln. of poly(methylvinylether-alt-maleic acid) sodium salt, **Polymer5**:Co=10) dissolved in 30 ml of deionized water ([Co] = 0.018 M). $NaBH_4$ (0.358 g, 9.369 mmol) was dissolved in 16.6 ml ([$NaBH_4$] = 0.550 M).

  TEM: D = 1.69 ± 0.51 nm.

- **Co6a:** was synthesized according to the general procedure using $CoCl_2{\cdot}6H_2O$ (0.226 g, 0.931 mmol) in presence of **Polymer6** (1.670 g of 50 wt% sln. of poly(methacrylicacid) sodium salt, **Polymer6**:Co=10) dissolved in 50 ml of deionized water ([Co] = 0.018 M). $NaBH_4$ (0.358 g, 9.369 mmol) was dissolved in 16.6 ml ([$NaBH_4$] = 0.550 M).

  TEM: D = 4.92 ± 1.15 nm.

- **Co6b (labeled in the paper as Co6):** was synthesized according to the general procedure using $CoCl_2{\cdot}6H_2O$ (0.226 g, 0.931 mmol) in presence of **Polymer6** (3.340 g of 50 wt% sln. of poly(methacrylicacid) sodium salt, **Polymer6**:Co=20) dissolved in 50 ml of deionized water ([Co] = 0.018 M). $NaBH_4$ (0.358 g, 9.369 mmol) was dissolved in 16.6 ml ([$NaBH_4$] = 0.550 M).

  TEM: D = 2.69 ± 0.58 nm.

  XRD: 46 º

  XPS: Co^0, 6%; Co^{2+}, 94%.

  TGA: Temperature ºC/Δ wt. % (attribution); 102/-3.17 (solvent); 253/-1.64 (polymer low); 900end/+0.17.

  ICP: Co, 73.7 wt%, B, 7.3 wt%; Co/B atom ratio, 2.02

  FTIR: 3428, 2925, 1619, 684 cm^{-1}.

  RAMAN: 664 cm^{-1}.

- **Co6c:** was synthesized according to the general procedure using $CoCl_2{\cdot}6H_2O$ (0.226 g, 0.931 mmol) in presence of **Polymer6** (6.680 g of 50 wt% sln. of poly(methacrylicacid) sodium salt, **Polymer6**:Co=40) dissolved in 50 ml of deionized water ([Co] = 0.018 M). $NaBH_4$ (0.358 g, 9.369 mmol) was dissolved in 16.6 ml ([$NaBH_4$] = 0.550 M).

  TEM: D = 1.91 ± 0.56 nm.

Table S1. summarizes the mean diameters and dispersions obtained for the series of **Co1-6** NPs as a function of the polymer:Co ratio.

Table S1. Summary of conditions used for the preparation of **Co1-6** by chemical reduction method.[a]

Entry	CoNPs (*)	Stabilizer	Polymer:Co[b]	Size, nm
1	**Co1a**	1	10	3.13 ± 1.58
2	**Co1b (Co1)**	1	20	2.64 ± 0.92
3	**Co1c**	1	40	2.11 ± 0.41
4	**Co2a**	2	10	6.73 ± 1.58
5	**Co2b (Co2)**	2	20	2.74 ± 0.75
6	**Co2c**	2	40	1.84 ± 0.74
7	**Co3a**	3	10	3.26 ± 1.12
8	**Co3b (Co3)**	3	20	2.83 ± 0.98
9	**Co3c**	3	40	1.67 ± 0.46
10	**Co4a**	4	10	4.12 ± 1.58
11	**Co4b (Co4)**	4	20	2.75 ± 0.57
12	**Co4c**	4	40	1.61 ± 0.67
13	**Co5a (Co5)**	5	1	2.55 ± 0.49
14	**Co5b**	5	5	2.07 ± 0.68
15	**Co5c**	5	10	1.69 ± 0.51
16	**Co6a**	6	10	4.92 ± 1.15
17	**Co6b (Co6)**	6	20	2.69 ± 0.58
18	**Co6c**	6	40	1.91 ± 0.56

[a] Conditions: 0.93 mmol of $CoCl_2.6H_2O$, 9.31mmol $NaBH_4$, water, 2h, rt. [b] Polymer:Co ratio based on mol of monomer units per mol of Co.* Labels used in the paper.

Figure S1. Mean diameter and size distributions for the series of CoNPs, **Co1-6** stabilized by **Polymers1-6** as a function of the polymer:Co ratio.

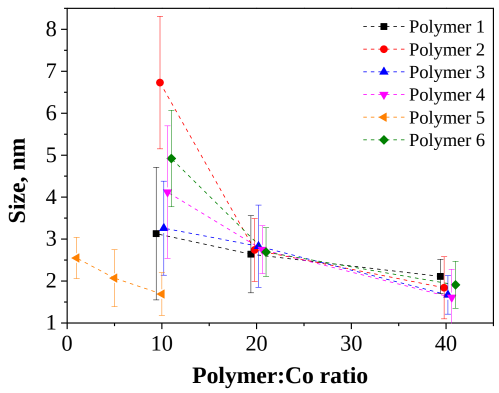

Figure S2. TEM micrographs and size histograms of **Co1-6** NPs.

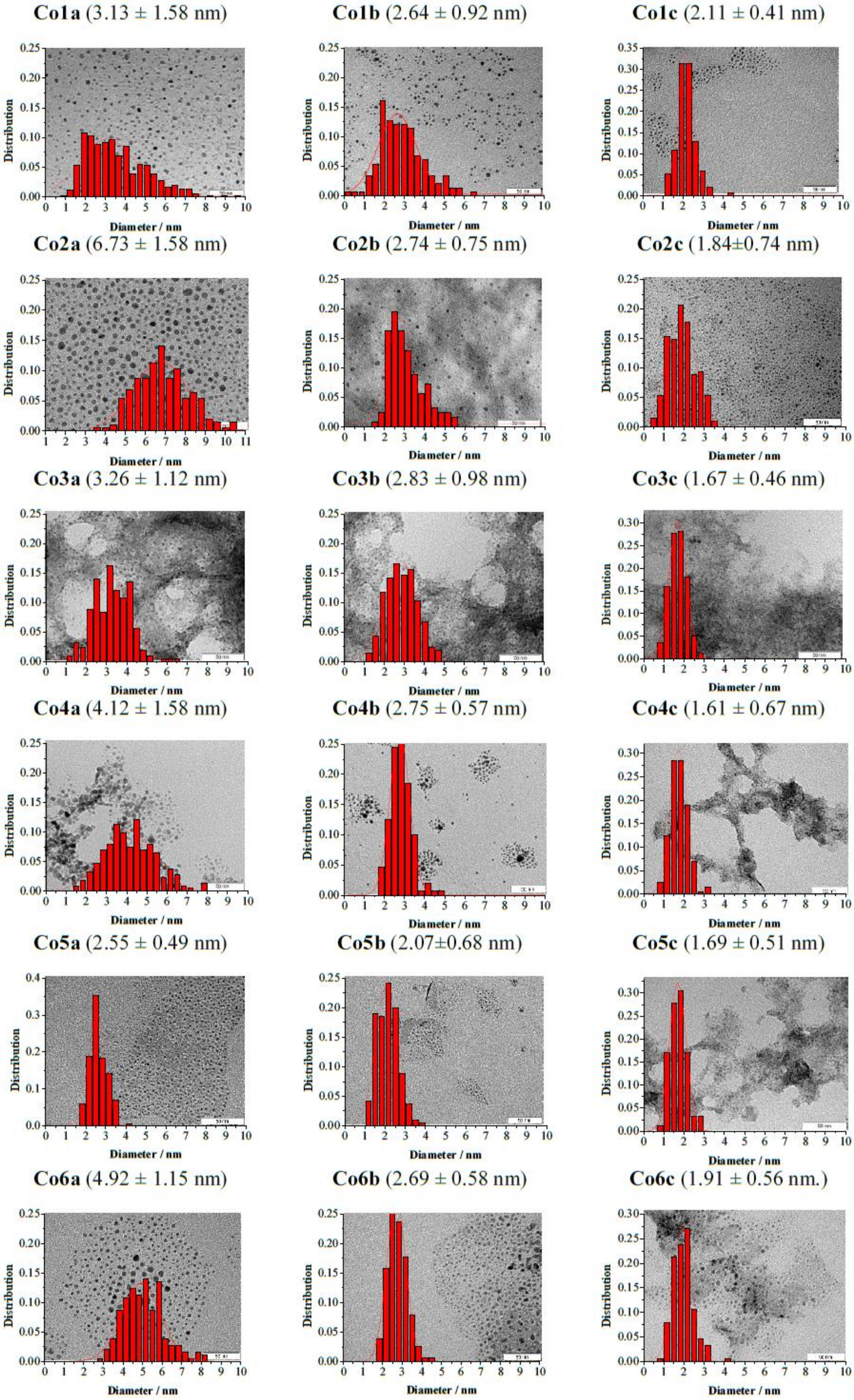

Figure S3. FTIR spectra of **Polymers1-6** (1) and its corresponding CoNPs, **Co1-6** (2)

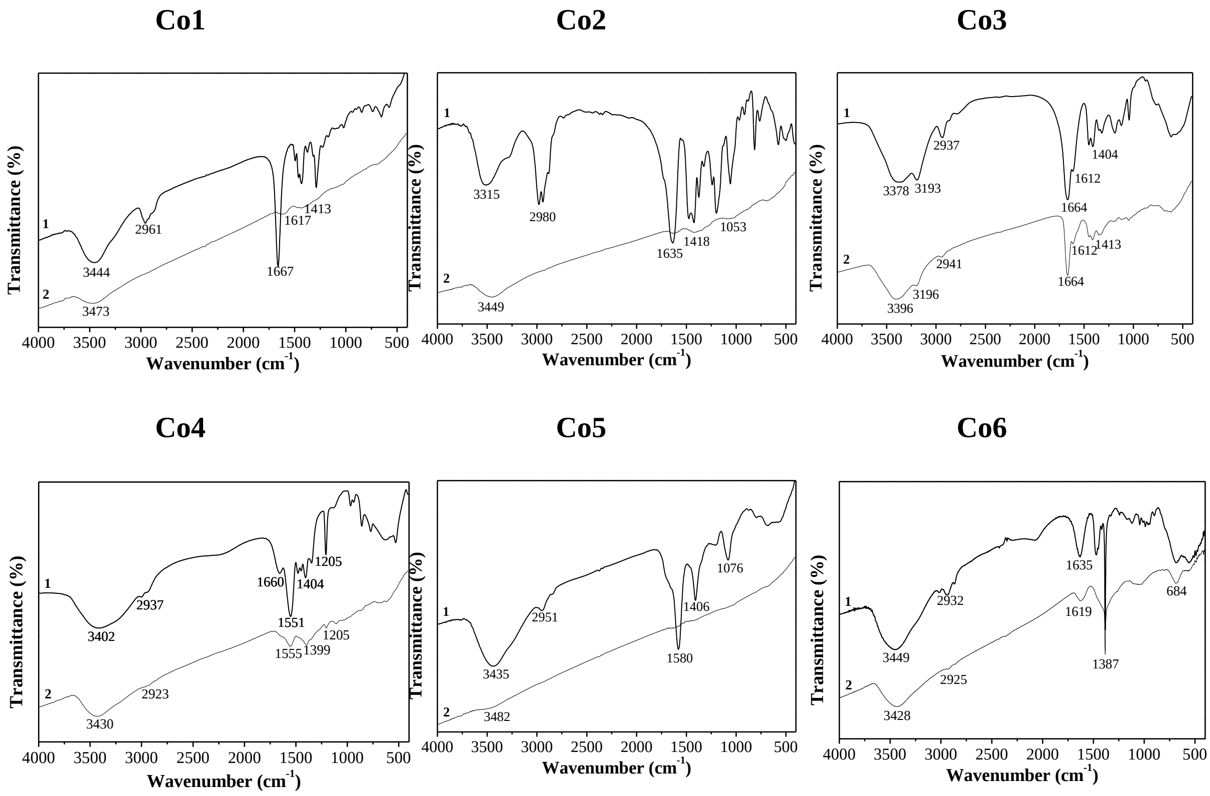

2. XPS analysis of the CoNPs before and after catalysis.

2.1. Evolution of the metallic cobalt content at the NPs surface after catalysis

Figure S4. Co 2p XPS spectra of **Co1-6** CoNPs (1) before and (2) after catalysis.

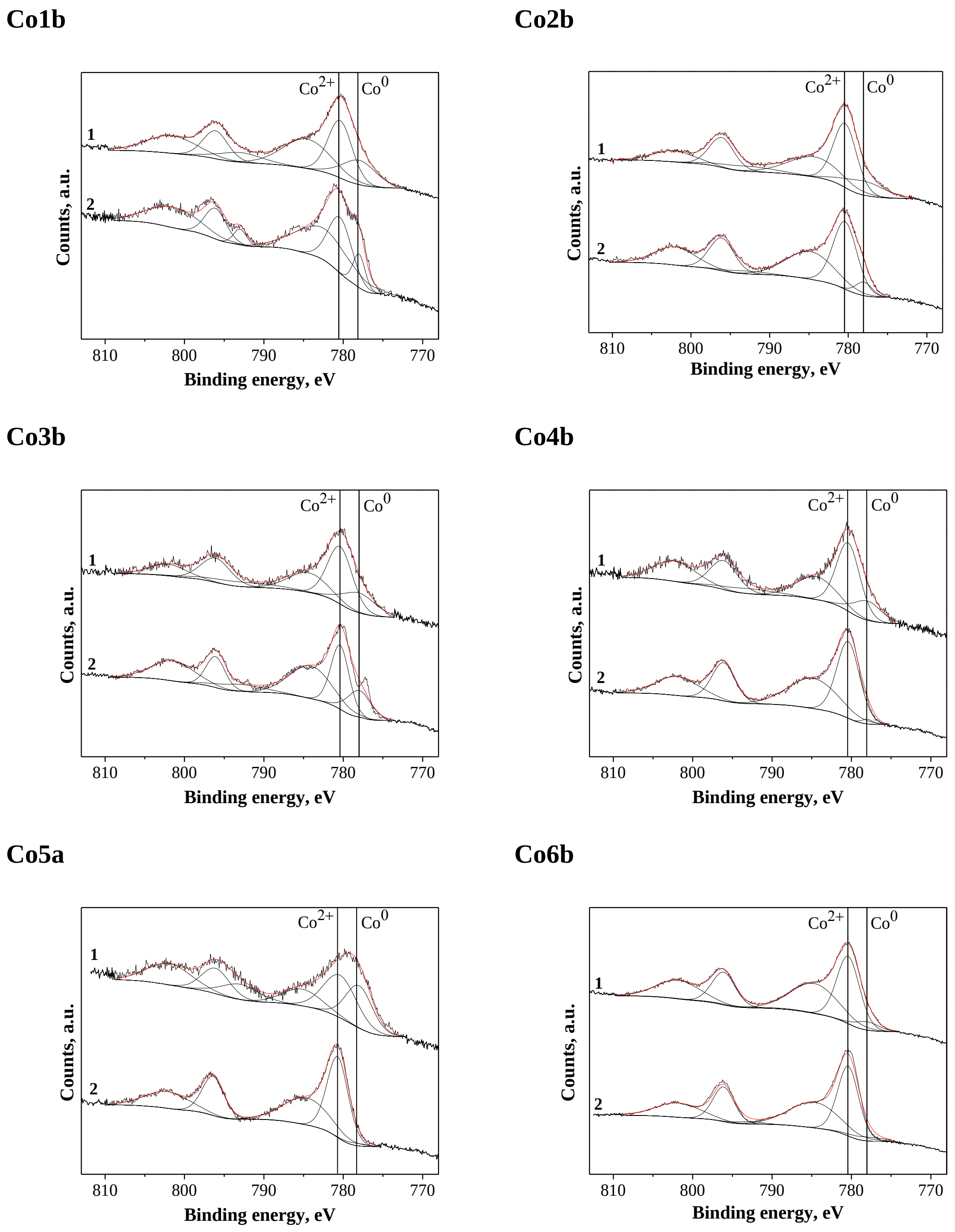

2.2. Evolution of the oxidation state of boron species at the NPs surface after catalysis

Figure S5. B 1s XPS spectra of **Co1-6** (1) before and (2) after catalysis.

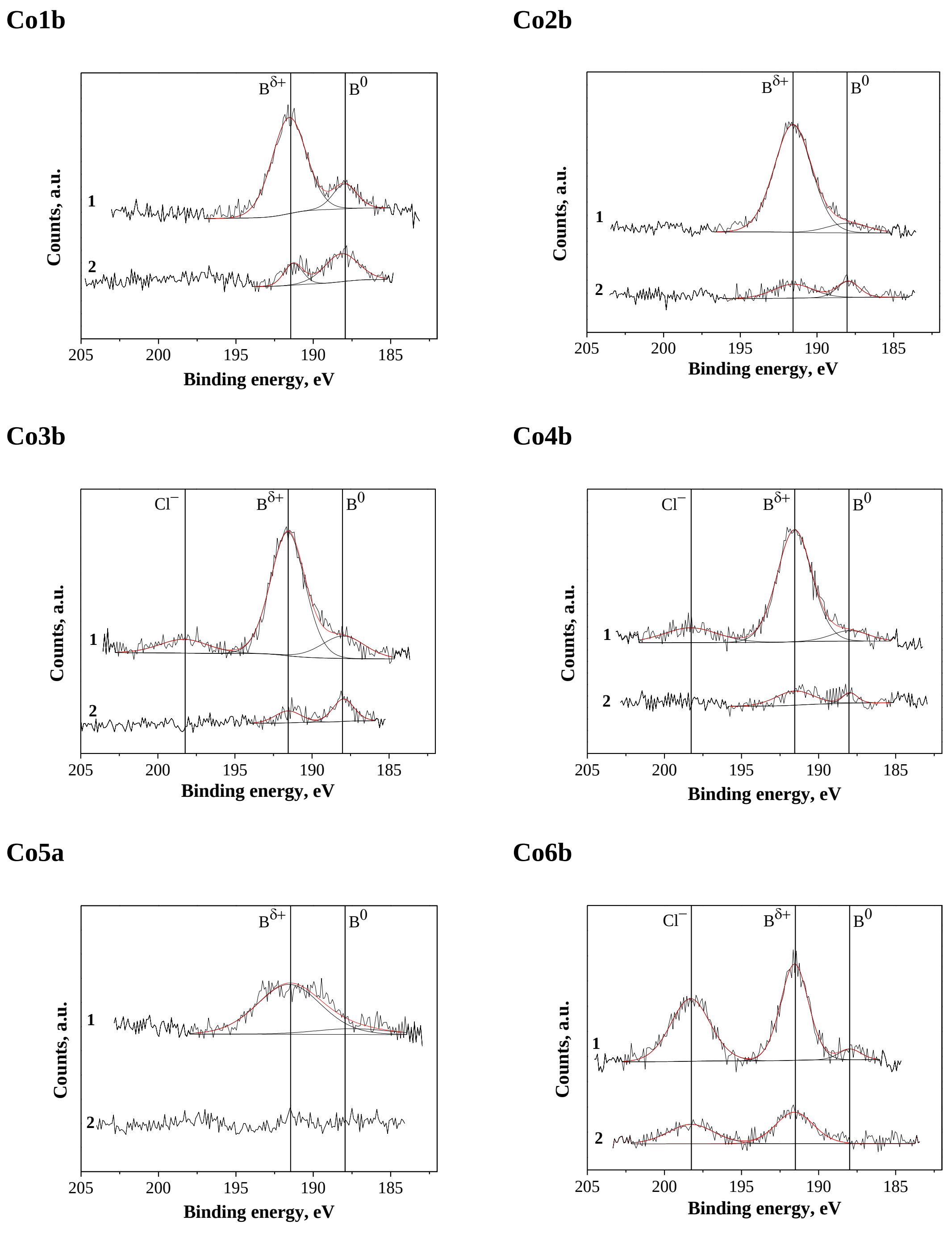

**Table S2.** Percentage of Co^0 and B^0 species in **Co1-6** NPs for fresh and used catalysis, from XPS spectra decomposition.[a]							
		Co^0			B^0		
Catalyst	Polymer	Fresh	Used	Δ%	Fresh	Used	Δ%
**Co1b**	1	37	24	-13	15	70	+55
**Co2b**	2	28	12	-16	9	39	+30
**Co3b**	3	31	35	+4	16	61	+45
**Co4b**	4	25	3	-22	8	23	+15
**Co5a**	5	67	3	-64	12	0	-12
**Co6b**	6	6	4	-2	4	0	-4

[a] Percentages corresponding to the Co $2p_{3/2}$ and B 1b spin orbit peaks.

**Table S3.** Elemental quantification by XPS of fresh **Co1-6** NPs.								
		Quantification regions (%Atom)						
NPs	Polymer	Na 1s	C 1s	N 1s	O 1s	Co 2p	B 1s	Co/B
**Co1b**	1		21		49	18	13	1.4
**Co2b**	2	2	40	1	40	9	9	1.0
**Co3b**	3	2	52	9	28	2	8	0.3
**Co4b**	4	2	59	8	26	2	4	0.5
**Co5a**	5	3	62		30	2	2	1.0
**Co6b**	6		27	2	44	14	13	1.1

**Table S4.** Elemental quantification by XPS of **Co1-6** NPs after AFTS.								
		Quantification regions (%Atom)						
NPs	Polymer	Na 1s	C 1s	N 1s	O 1s	Co 2p	B 1s	Co/B
**Co1b**	1		55	2	32	9	2	4.3
**Co2b**	2		41	1	42	16	1	20.6
**Co3b**	3		51	1	36	12		∞
**Co4b**	4		34	1	46	18	1	19.7
**Co5a**	5		62	0	33	4		∞
**Co6b**	6		29		51	18	3	6.3

2.3. Complementary XPs analysis (full XPS and C 1s) of the fresh catalysts.

Figure S6. (a) Full XPS and **(b)** C1s XPS spectra of **Co1-6** NPs.

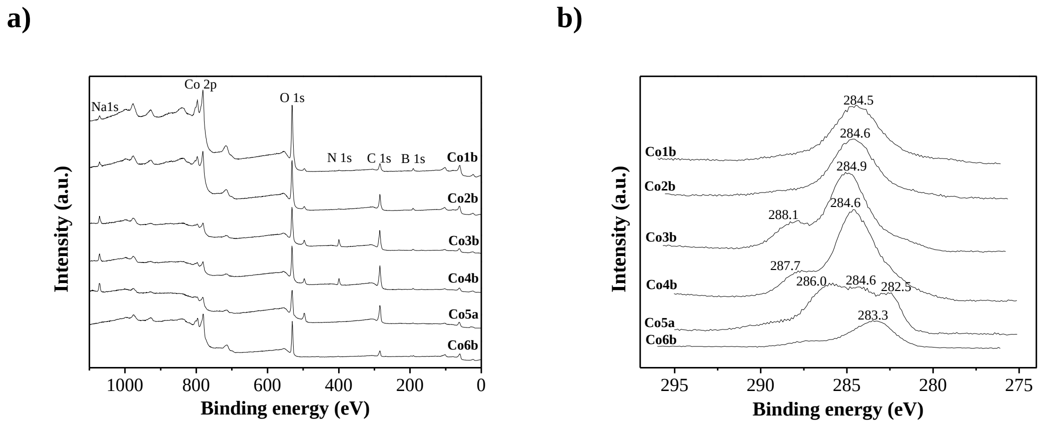

3. TEM analysis of the CoNPs after catalysis.

Figure S7. TEM micrographs and size histograms of **Co1-6** NPs after AFTS reactions.

Co1b: 3.28 ± 1.17 nm

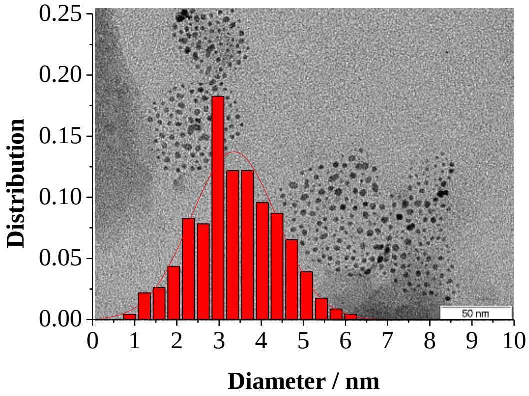

Co2b: 1.5 ± 0.5 nm

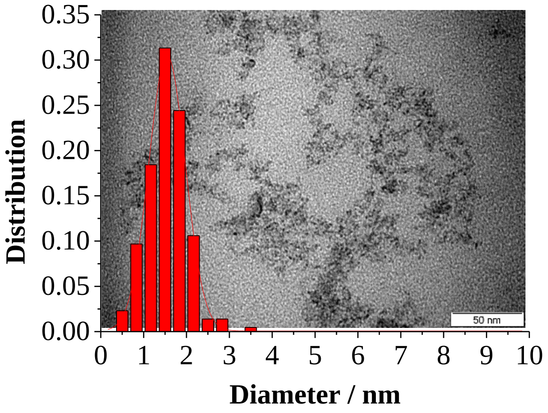

Co3b: 11.6 ± 4.9 nm

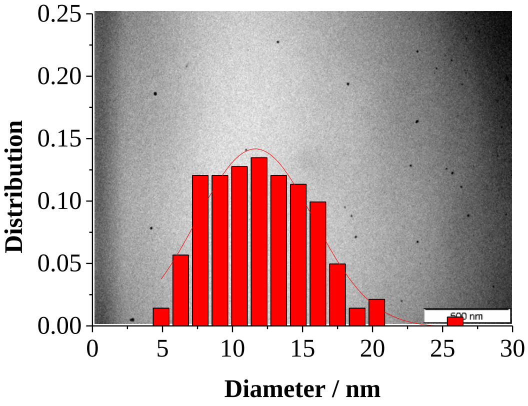

Co4b: 2.2 ± 0.7 nm

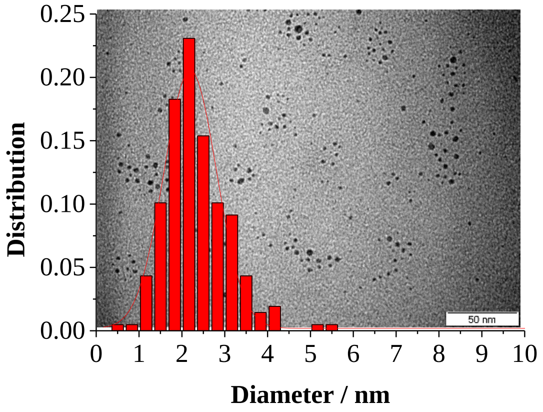

Co5a: 3.7 ± 1.3 nm

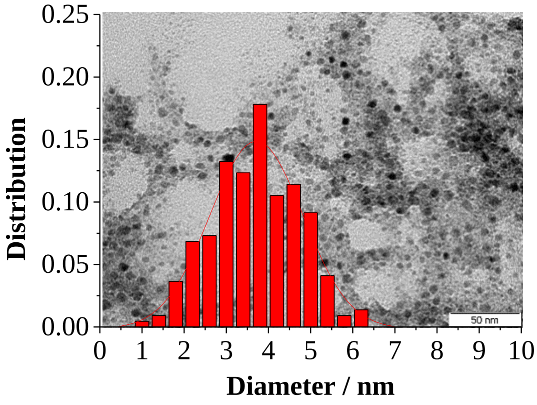

Co6b: 2.8 ± 1.0 nm

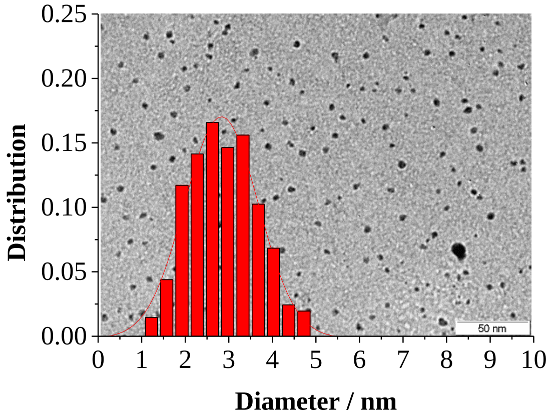

**Table S5.** Variation of the cobalt particle size after AFTS				
Parameter studied	Value	Catalyst	Fresh NPs	Used NPs
Polymer effect	1	**Co1b**	2.6 ±0.9	3.28 ±1.17
	2	**Co2b**	2.7 ±0.7	1.5 ±0.5
	3	**Co3b**	2.8 ±1.0	11.6 ±4.9
	4	**Co4b**	2.7 ±0.6	2.2 ±0.7
	5	**Co5a**	2.5 ±0.5	3.7 ±1.3
	6	**Co6b**	2.7 ±0.6	2.8 ±1.0

4. FTIR spectra of the CoNPs after catalysis.

Figure S8. FTIR spectra of **Polymers1-6** (1) and its corresponding CoNPs, **Co1-6** before (2) and after catalysis (3)

Co1a

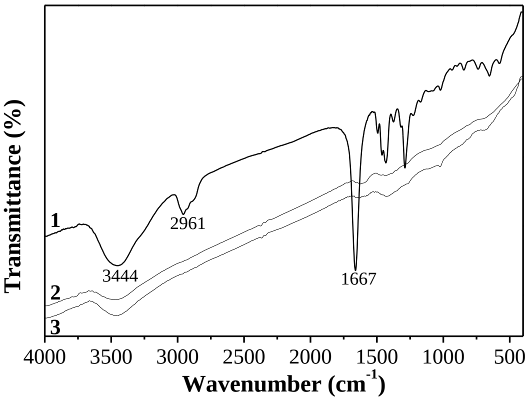

Co2b

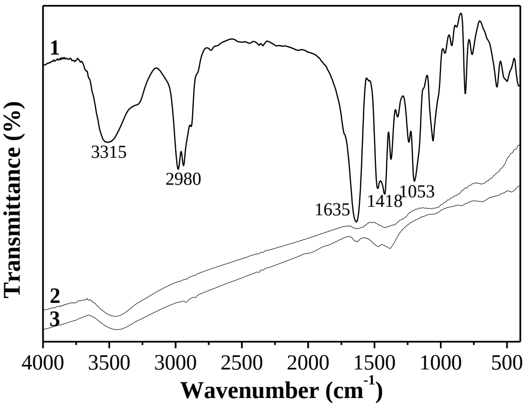

Co3b

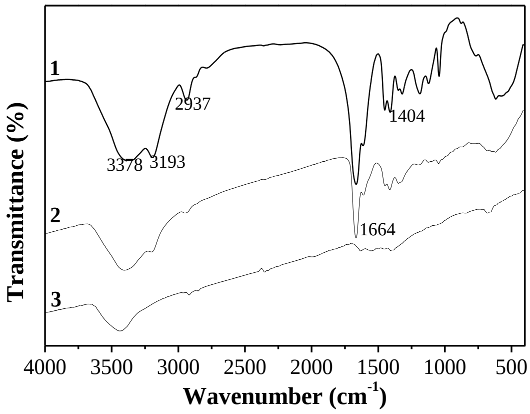

Co4b

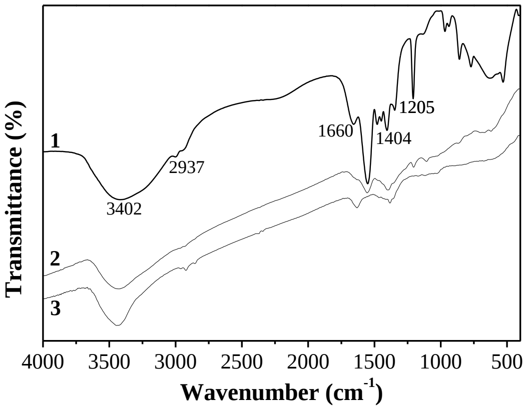

Co5a

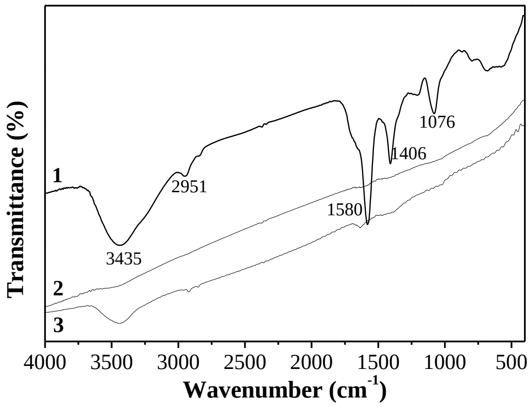

5. Aqueous phase Fisher-Tropsch synthesis (AFTS) data

Table S6. Aqueous phase Fischer-Tropsch synthesis catalyzed by **Co1-6** evaluating the effect of the polymer stabilizer on the NPs.[a]

E.	Catalyst	Polymer	Cobalt time yield, h^{-1} (+CO_2)[b]	Selectivity, Wt% CO_2	CH_4	C_{2-4}	C_{5-12}	α
1	**Co1**	1	0.026 (0.029)	23	20	33	24	0.59
2	**Co2**	2	0.021 (0.027)	43	19	24	14	0.66
3	**Co3**	3	0.033 (0.040)	42	12	30	16	0.60
4	**Co4**	4	0.002 (0.029)	96	2	1	1	0.65
5	**Co5**	5	0.061 (0.068)	26	19	40	15	0.50
6	**Co6**	6	0.051 (0.058)	28	48	16	8	0.53

[a] Conditions: 0.949 mmol Co, 30 bar H_2/CO/Ar (2:1:0,15); 66ml water (100 ml autoclave), 1000rpm, 180 ℃, 12h [b] metal time yield = mol of CO converted to hydrocarbon products per mol of Co, per unit of time (in parenthesis the CO converted to CO_2 is included)

Table S7. Aqueous phase Fischer-Tropsch synthesis catalyzed by **Co1-6** evaluating the effect of the polymer stabilizer on the NPs.[a]

E.	Catalyst	Polymer	O/P ratio O/P C_{2-12}	O/P C_{2-4}	O/P C_{5-12}
1	**Co1b**	1	0.79	1.33	0.28
2	**Co2b**	2	0.98	1.38	0.35
3	**Co3b**	3	1.26	1.90	0.43
4	**Co4b**	4	0.31	0.71	0.00
5	**Co5a**	5	1.32	1.50	0.79
6	**Co6b**	6	0.92	1.39	0.37

[a] Conditions: 0.949 mmol Co, 30 bar H_2/CO/Ar (2:1:0,15); 66ml water (100 ml autoclave), 1000rpm, 180 ℃, 12h

Figure S9. ASF distributions of **Co1-6** nanocatalysts.

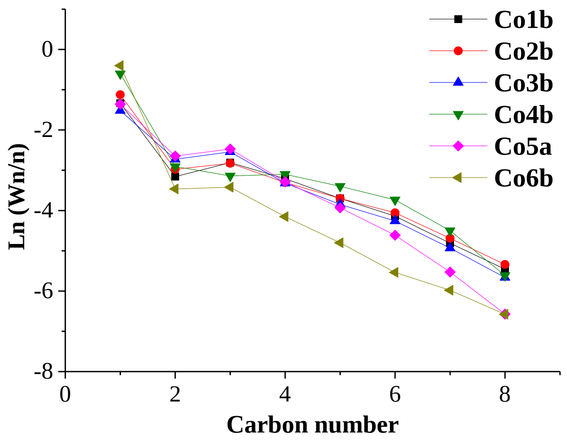
Co1b
Co2b
Co3b
Co4b
Co5a
Co6b
Ln (Wn/n)
Carbon number
0
-2
-4
-6
-8
0
2
4
6
8
